# The efficacy and safety of leucine-enriched essential amino acids in knee osteoarthritis patients: A randomized controlled trial

**DOI:** 10.1097/MD.0000000000038168

**Published:** 2024-05-10

**Authors:** Seung-Jun Park, Chang Hyun Nam, Hye Sun Ahn, Taehyun Kim

**Affiliations:** a Himchan and University Hospital Sharjah Spine and Joint Centre, University Hospital Sharjah, Sharjah, United Arab Emirates; b Joint and Arthritis Research, Himchan Hospital, Seoul, Korea; c Department of Orthopedic Surgery, Mok-dong Himchan Hospital, Seoul, Korea.

**Keywords:** essential amino acids, LEAA, leucine, osteoarthritis, protein supplementation

## Abstract

**Background:**

Supplementation with leucine-enriched essential amino acids (LEAAs) has shown efficacy in the recovery of muscle injury and activation of muscle synthesis. Muscle function in knee osteoarthritis is a crucial factor for managing pain and preserving ambulatory function. However, the efficacy and safety of LEAAs supplementation in patients with knee osteoarthritis have not been evaluated.

**Methods:**

In this prospective analysis, we evaluated the efficacy and safety of supplementation with 12 g of LEAAs daily for 8 weeks in knee-symptomatic osteoarthritis patients. For assessing the efficacy, clinical pain, calf circumference, and disability were assessed using questionnaires (visual analog scale, Knee Society Score, and 36-item short form survey [SF-36]), laboratory analyses (total protein and albumin), and radiologic study (dual-energy X-ray absorptiometry [DEXA]) for muscle and bone density. To evaluate safety, generalized or localized protein allergic reactions, complete blood count, liver and kidney function, and serum glucose were measured.

**Results:**

Sixty-five participants, categorized into the experimental (n = 32) and control (n = 33) groups, were included in this 8-week trial from March 2022 to July 2022. A significantly higher efficacy was observed in the experimental group than in the control group, as indicated by muscle density in the DEXA scan (*P* = .001) and SF-36 (*P* < .001). The safety evaluation revealed no related generalized or local protein allergy. Hematological findings, serum glucose, and kidney and liver toxicity were not significantly different between the groups.

**Conclusion:**

Supplementation with leucine-enriched proteins is safe and efficacious in the improvement of muscle density and quality of life.

## 1. Introduction

With the aging of society, musculoskeletal disorders are becoming a major issue for individuals and societies. Among them, knee osteoarthritis is one of the main conditions affecting quality of life, as the knee plays a major role in supporting body weight. Treatment for knee arthritis vary depending on the stage.^[1]^ In its early stages, knee arthritis can be treated by conservative measures, including nonsteroidal anti-inflammatory medications and intra-articular injection.^[2,3]^ In advanced stages, surgical interventions such as total knee arthroplasty can be considered.^[4]^ Moreover, pain control, alleviation of muscle stiffness, and muscle strengthening with nutritional supplementation^[5]^ and regular exercise are the focus of management in all stages. In knee osteoarthritis patients, the muscle quality and function of the lower limbs are important. For restoring function and compensating the muscle, various physical exercises and nutrient supplements have been attempted.^[6]^ Aquatic exercise,^[7]^ various forms of physiotherapy,^[8,9]^ diet change for weight loss,^[10,11]^ glucosamine, and chondroitin sulfate^[12,13]^ are frequently prescribed by primary physicians. With regards to nutritional support, knee joints consist of cartilage, bone, and muscles, but nutritional research has mainly focused on cartilage and bones, rather than on supporting muscles. In old age, muscle volume loss due to sarcopenia and the advancement of osteoarthritis are closely interrelated and affect the quality and span of life.^[12–15]^ Recently, protein supplementation, especially with leucine-enriched essential amino acids (LEAAs), has shown efficacy in helping muscle recovery after exercise and stimulating muscle synthesis.^[16,17]^ Protein administration in older individuals is usually based on vegetable sources such as soy protein; however, possibly insufficient leucine content has been a concern, as it is considered essential for muscle synthesis and recovery.^[14,15]^ Supplementation with LEAAs after total knee replacement showed efficacy in preventing loss of muscle volume.^[16,17]^ However, the early stage before surgery has not been evaluated, and a comprehensive analysis of the patient condition and the safety of LEAAs administration has not been conducted to date. Thus, this study aimed to verify the safety of LEAA administration and its efficacy in pain control and improving quality of life in a sample of early knee arthritis patients.

## 2. Methods

### 2.1. Study design

This single-center prospective comparative analysis employed an alternative random allocation method, was approved by the institutional review board (116655-01-202203-01), and was registered in the national clinical trial system (KCT0007363) before trial. All procedures adhered to the Declaration of Helsinki. All participants were recruited in the outpatient clinic of orthopedics, Mok-dong Himchan Hospital, Yangcheon-gu, Seoul, Republic of Korea, and all participants gave their written consent. The sample size was calculated using G*power, 3.1.9.7 (Aichach, Germany), and the groups (experimental and control) were randomized using a block randomization method before allocation. The allocation was done in a parallel 1:1 method. The experimental group took LEAAs (leucine, isoleucine, phenylalanine, threonine, valine histidine, methionine, lysine, and tryptophan, and a combination of whey protein and soy protein; S.chan Protein®, Chun-chon, Kangwon, Korea) twice daily. A total of 25 g of protein powder was added to water (120 mL) and taken for 8 weeks regardless of meals. The experimental and control groups without intervention were evaluated initially and weekly for 8 weeks. All laboratory and radiologic assessments as well as the questionnaires were performed before and after the administration of the supplements in both the experimental and control groups.

### 2.2. Inclusion and exclusion criteria

All data processes were anonymized, and information on sex, age, and body profile (height, weight, and body mass index) was collected before and after the trial. History of allergies, underlying diseases, vital signs (blood pressure, body temperature, respiratory rate, and pulse rate), and medication history were recorded. Knee arthritis grade was evaluated by magnetic resonance imaging and classified based on the International Cartilage Regeneration and Joint Preservation Society Classification (ICRS).^[18]^ In the 3 months preceding the trial and throughout its duration, any supplementary medication and physiotherapy were restricted. The inclusion criteria were as follows: knee osteoarthritis diagnosed by clinical history and plain anterior-posterior radiograph on standing position and magnetic resonance imaging, and normal or mild systemic disease (American Society of Anesthesiologists classification of physical status between 1 and 2). The exclusion criteria were other joint degenerative diseases (spinal stenosis, and hip or ankle osteoarthritis), nonambulatory status, and infection or tumorous conditions.

### 2.3. Efficacy evaluation criteria

**Figure 1.**
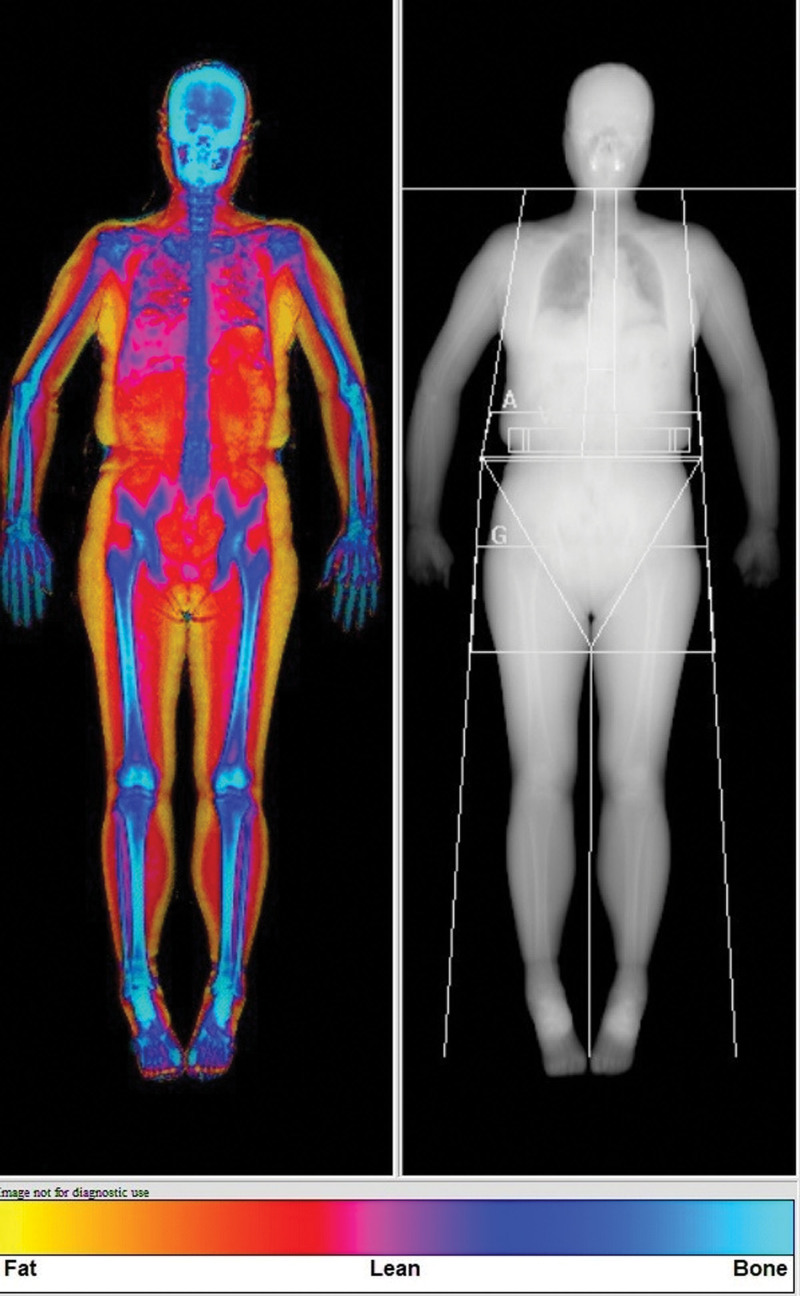
Example of dual-energy X-ray absorptiometry for muscle (lean) mass and bone density.

Considering the lifestyle and disability characteristics associated with knee osteoarthritis and the diverse study population, we utilized 3 questionnaires and conducted 7 examinations. Prior to and following the supplementation period, pain levels were quantified using a visual analog scale (0–10). Additionally, physical function, floor life, and socio-emotional function were assessed using the Korean knee score (KKS, score of 0–100).^[19]^ To gauge osteoarthritis related scores for pain, stiffness, physical function, and overall disability, we employed the Western Ontario and McMaster Universities Osteoarthritis Index (WOMAC).^[20]^ General health status was assessed using the short form 36 health survey (SF-36).^[21]^ Muscle volume and bone marrow density were assessed using a dual-energy X-ray absorptiometry machine (DEXA, HorizonR, Hologic, Marlborough, Massachusetts, United States, Fig. 1). Calf muscle size was measured using a tape measure in the sitting position and barefoot. Walking speed (km/h), chair standing exercise (/min), and grip strength (kg, Jamal dynamometer) were assessed as indicators of physical performance. Laboratory tests were compared before and after supplementation. The results of a blood test from the antecubital vein, serum protein, and albumin were also compared.

### 2.4. Safety evaluation criteria

For the evaluation of generalized protein allergic reactions, vital signs (blood pressure, body temperature, respiratory rate, and pulse rate) were determined first after 30 minutes of administration and then at the checkup visits at weeks 1 and 8. During the visits, physical examinations for skin allergic reactions were also performed. During the trial period, a physician monitored all adverse reactions by phone.

### 2.5. Outcome measures

Serum hemoglobin was monitored, and a differential study for hematologic reactions, glucose, and insulin were performed to assess blood sugar control. The levels of aspartate transaminase/alanine aminotransferase (ALT) and alkaline phosphatase (ALP) were determined as a measure of hepatotoxicity. Blood urea nitrogen/creatinine (BUN/Cr) was measured for renal toxicity. All subjective and objective adverse reactions were classified according to severity and degree of association with LEAA supplementation from mild to severe according to the World Health Organization Adverse Reaction Terminology.^[22]^

### 2.6. Statistical analysis

All numerical results, including age, serum markers, pain scale, and questionnaire scores between the groups, were compared using independent t-tests following a normality test. The differences before and after the administration were analyzed using paired Student t-tests. Categorical variables, including sex and allergic reactions, were compared using the chi-square test. Data were processed using the Statistical Package for Social Sciences (Version 24.0, Chicago, IL), and a *P* value of <.05 was considered statistically significant.

## 3. Results

### 3.1. Patient demographic information

**Figure 2.**
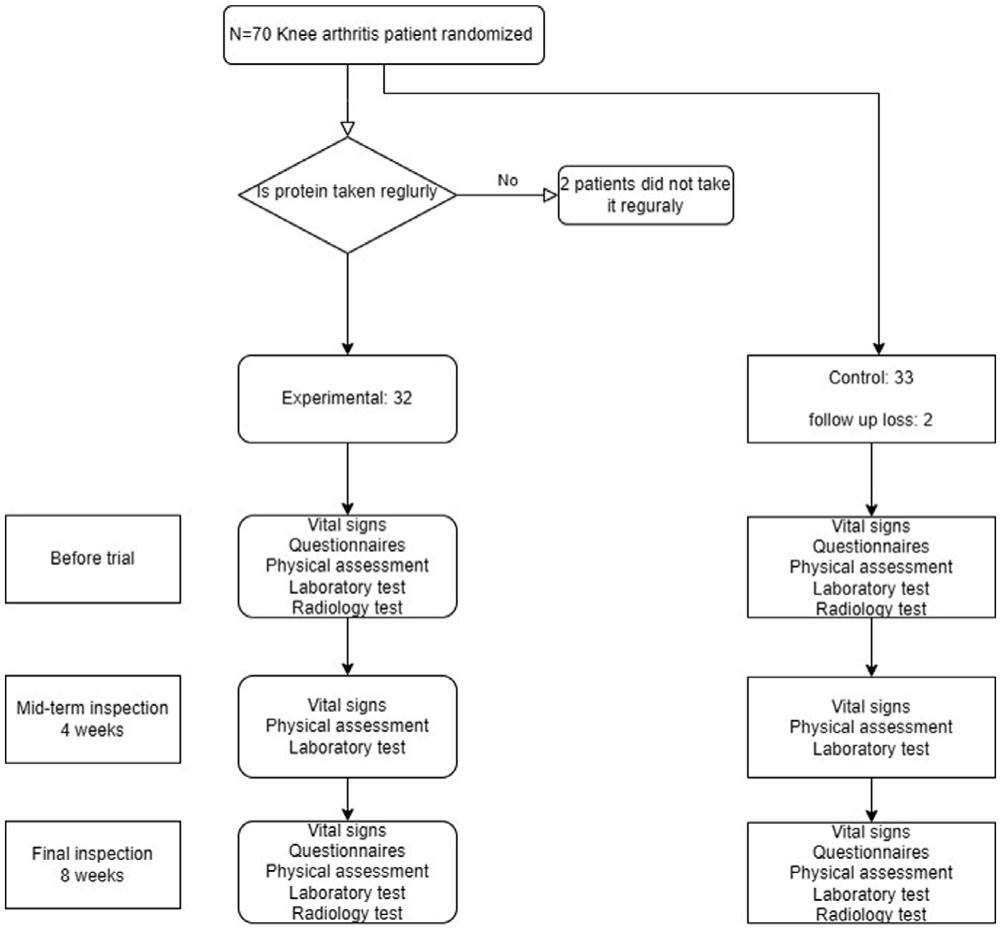
Recruitment of subjects, group allocation, and follow-up schedules.

A total of 70 participants were recruited. Among them, 3 patients were excluded because of insufficient or irregular intake, and 2 patients were excluded because they were lost to follow-up. From March 2022 to June 2022, a total of 65 patients were included in the study—32 of which were allocated to the LEAA (experimental) group and 33 to the control group—and followed up (Fig. 2). There were differences between the groups in the demographic characteristics of patients, including in age (*P* = .73), sex (*P* = .44), and body mass index (LEAAs, *P* = .82; control, *P* = .29). The ICRS grade in the experimental group was 1.8 ± 1.0 in both the right and left side, while the corresponding values in the control group were 1.8 ± 0.8 (left) and 1.8 ± 0.9 (right), with no significant difference between the groups (Table 1).

**Table 1:** Participant demographics of the leucine-enriched protein group and the control group.

Criteria	Experimental			Control			*P* value
Sex (Male: Female)	12:20			11:22			.73
Age, yr (n)	63.2 ± 7.4 (51–78)			64.6 ± 7.2 (51–78)			.44
Height (cm)	160.38 ± 7.77			160.64 ± 7.83			.45
Weight (kg)	63.34 ± 9.33	63.41 ± 9.51	0.979	61.52 ± 10.21	61.17 ± 10.04	0.889	.23
ICRS grade (Left)	1.8 ± 1.0			1.8 ± 0.8			.72
ICRS grade (Right)	1.8 ± 1.0			1.8 ± 0.9			.41
Body mass index	24.6 ± 3.1 (Before)	24.6 ± 3.0 (After)	0.816	23.7 ± 2.6	23.6 ± 2.6	0.288	.35

ICRS = International Cartilage Regeneration and Joint Preservation Society Classification.

*P ≤ .05.

### 3.2. Efficacy of LEAAs supplementation

The detailed efficacy of LEAA supplementation and the comparison between the groups are summarized in Table 2.

**Table 2:** Summary statistics of efficacy in the leucine-enriched protein group and control group.

		Experimental (LEAAs)			Control			Confidence interval	*P* value
Physical assessment	Pain (VAS)	4.1 ± 1.2	3.4 ± 1.0	0.002	3.0 ± 1.0	2.6 ± 0.9	0.017*	0.48–1.58	.43
	Calf circumference (cm)	35.4 ± 2.9	35.8 ± 2.7	0.028*	34.9 ± 2.4	34.9 ± 2.4	0.728	−0.32 to 2.18	.08
	Grip power (kg)	29.1 ± 7.4	29.6 ± 8.1	0.267	28.9 ± 7.5	29.0 ± 7.4	0.710	−3.30 to 4.35	.50
Questionnaires	Knee evaluation score*	85.6 ± 9.6	86.4 ± 8.9	0.238	87.8 ± 8.9	88.0 ± 8.9	0.473	−0.12 to 0.12	.52
	Osteoarthritis score (WOMAC)	20.3 ± 8.9	16.8 ± 7.7	<0.0001***	16.3 ± 14.4	13.5 ± 6.1	0.180	−0.14 to 6.76	.77
	Quality of life (SF-36***)	72.5 ± 11.0	77.8 ± 7.2	<0.0001***	80.9 ± 5.5	81.8 ± 5.1	0.010**	−7.16 to 0.97	<.001*
Radiology	Muscle (DXA, kg/m^2^)	5.77 ± 1.0	5.93 ± 1.0	<0.0001***	5.72 ± 1.0	5.69 ± 0.9	0.239	−0.25 to 0.72	<.001*
	Bone marrow density	−1.8 ± 1.4	−1.7 ± 1.6	0.696	−2.3 ± 1.2	−2.5 ± 1.2	0.018*	0.43–1.43	.38
Hematology	Total protein (g/dL)	6.9 ± 0.4	6.9 ± 0.4	0.111	6.8 ± 0.3	6.9 ± 0.3	0.061	−0.14 to 0.17	.01*
	Albumin(g/dL)	4.3 ± 0.2	4.2 ± 0.2	0.002**	4.3 ± 0.2	4.3 ± 0.2	0.756	−0.18 to 0.02	.05*

DXA = dual X-ray absorptiometry, KSS = knee society score, LEAAs = leucine-enriched essential amino acids, SF-36 = short form 36 health survey, WOMAC = Western Ontario and McMaster universities osteoarthritis index

* *P* ≤ .05.

** *P* ≤ .01.

*** *P* ≤ .001.

**Figure 3.**
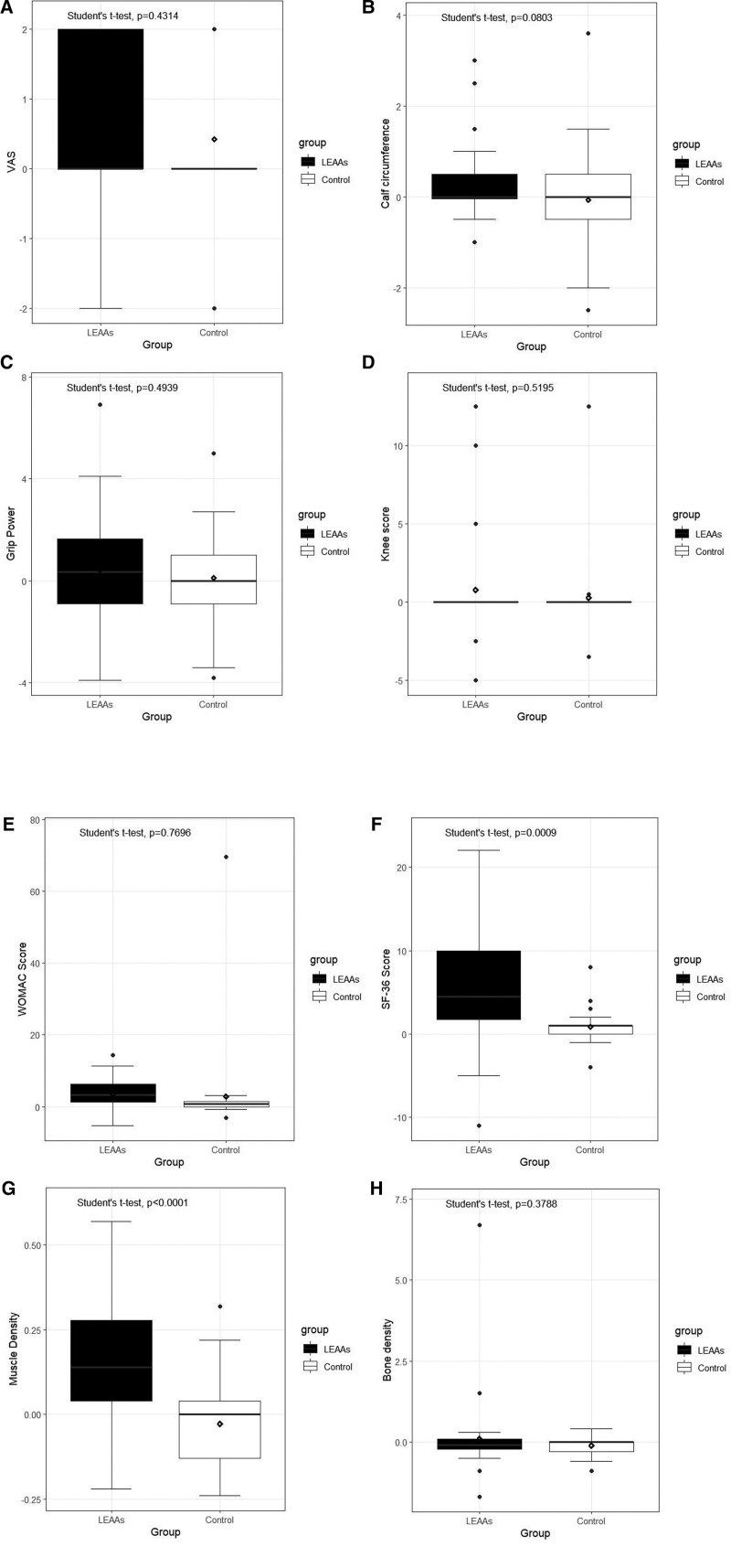
Comparison of efficacy criteria in the leucine-enriched protein group and control group. (A) VAS for pain assessment. (B) Calf circumference for measuring muscle mass. (C) Grip strength (kg) for muscle power. (D) Knee Society Scoring Questionnaire (E). WOMAC. (F) SF-36. (G) Muscle density improvement. (H) Bone density improvement. LEAAs = leucine-enriched essential amino acids, SF-36 = 36-item short form survey, VAS = visual analog scale, WOMAC = Western Ontario and McMaster Universities Osteoarthritis Index.

In the physical assessment, both the LEAA and control groups showed pain improvement (*P* = .002, *P* = .02), and calf circumference improved only in the LEAA group (*P* = .03). However, the differences in pain (Fig. 3A) and calf circumference (Fig. 3B) were not significant between the groups (*P* = .43). Grip strength did not improve in any of the groups (*P* = .27 and *P* = .71, respectively, Fig. 3C). There were also no significant improvements in the osteoarthritis score or the Knee Society Score in any group (Fig. 3D), but the WOMAC score improved significantly in the LEAA group (*P* < .001). However, this result was not superior to that of the control group (Fig. 3E, *P* = .77). The general health and quality of life questionnaire (SF-36) showed significantly better results in the LEAA group than in the control group (*P* < .001, Fig. 3F). Radiologically, muscle density increased by about 0.15 kg/m^2^ in the LEAA group. In contrast, the control group did not improve after the intervention (*P* < .001, Fig. 3G). Bone density did not change after supplementation (*P* = .38, Fig. 3H). The laboratory test showed that total protein did not change in the LEAA group (*P* = .11) or in the controls (*P* = .06). Albumin decreased in the LEAA group only (*P* = .002) and did not change in the control group (*P* = .76), but the difference was within the normal range.

### 3.3. Safety of LEAAs supplementation

**Figure 4.**
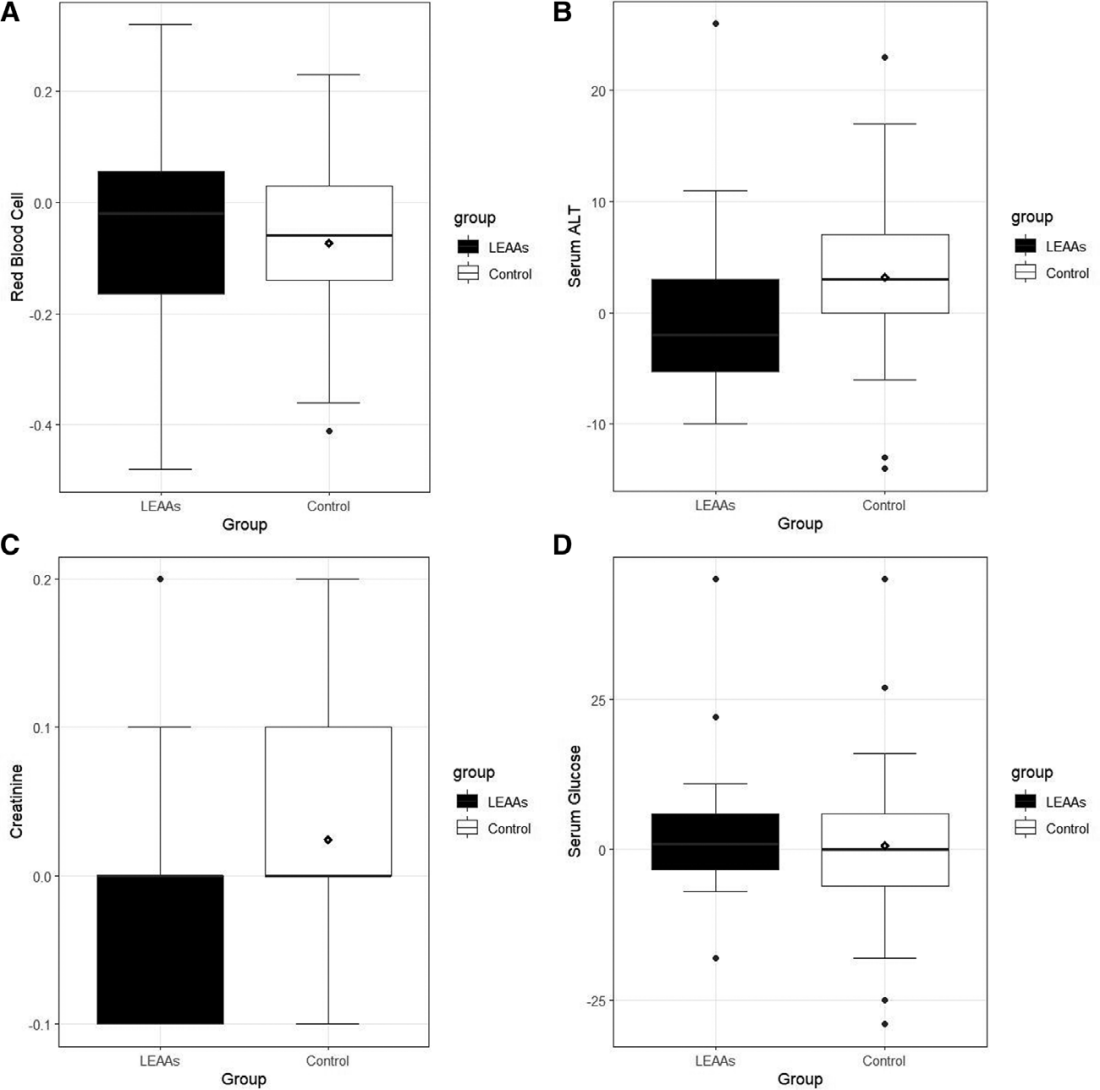
Comparison of safety criteria after leucine-enriched protein supplementation. (A) Red blood cells. (B) liver enzyme (ALT). (C) Kidney function test (creatinine). (D) Blood sugar (serum glucose). ALT = alanine aminotransferase, LEAAs = leucine-enriched essential amino acids.

In this study, we found no generalized or local allergic or adverse reactions. All the laboratory tests for kidney, liver, and blood sugar are summarized in Table 3. In the hematological test, only the control group showed a decrease in red blood cell count (*P* = .01); however, the difference between the groups was not significant (*P* = .48, Fig. 4A). In both groups, white blood cells for inflammatory reaction and autoimmune reaction did not change in any group between before and after the treatment, and the difference between the groups was also not significant. In the liver function test, only the control group showed increments in ALT, and ALP (*P* < .001, Fig. 4B and *P* = .019, respectively), and the differences between the groups were significant (*P* < .001 and *P* = .05), although the values within the normal range (ALT 10–40 International units (IU)/L, ALP 40–111 IU/L). In the kidney function test, BUN increased in the LEAA group (*P* < .001), but the comparison with the control group showed no significant differences (*P* = .57). The change in Cr in the LEAA and control groups was not significant (*P* = .26 and *P* = .073, respectively, Fig. 4C). Blood sugar changed (*P* = .53) in both the LEAA and control groups, as indicated by a slight decrease in serum glucose after supplementation; however, this change was not statistically significant (*P* = .18 and 0.78, respectively, Fig. 4D).

**Table 3:** Summary of safety criteria in the leucine-enriched protein group and control group.

		Experimental			Control			*P* value
Hematology	Red blood cells (g/dL)	4.6 ± 0.4	4.6 ± 0.4	0.242	4.5 ± 0.3	4.4 ± 0.4	0.010**	.48
	White blood cells (cells*10^3^/µL)	6.2 ± 1.3	6.2 ± 1.3	0.860	7.0 ± 2.0	6.8 ± 2.2	0.280	.50
	Platelets (cells*10^9^/L)	245.4 ± 49.2	241.5 ± 52.6	0.380	258.0 ± 50.5	246.9 ± 54.0	0.058	.32
Liver function	Aspartate transaminase (IU/L)	28.9 ± 12.9	28.1 ± 9.9	0.470	23.8 ± 5.1	28.2 ± 5.7	<0.0001***	<0.001***
	Alanine aminotransferase (IU/L)	26.2 ± 8.7	25.5 ± 11.2	0.619	22.2 ± 9.8	25.4 ± 9.6	0.019*	<.05*
	Alkaline phosphatase (IU/L)	72.5 ± 11.0	77.8 ± 7.2	<0.0001***	80.9 ± 5.5	81.8 ± 5.1	0.010**	.32
Kidney function test	Blood urea nitrogen (mg/dL)	5.77 ± 1.0	5.93 ± 1.0	<0.0001***	5.72 ± 1.0	5.69 ± 0.9	0.239	.57
	Creatinine (mg/dL) tests (µmol/L)	1.0 ± 0.2	0.9 ± 0.2	0.258	0.9 ± 0.2	1.0 ± 0.2	0.073	.04*
Blood sugar	Serum glucose (mmol/L)	101.8 ± 16.5	99.2 ± 15.1	0.180	97.2 ± 12.1	96.5 ± 9.5	0.781	.54
	Hemoglobin A1c (%)		5.8 ± 0.6		5.7 ± 0.5			.41

IU = international units.

* *P* ≤ .05.

** *P* ≤ .01.

*** *P* ≤ .001.

## 4. Discussion

We hypothesized that supplementation with LEAAs in osteoarthritis patients can increase serum protein level, increase muscle mass physically, and improve disability and knee function. The results showed that, in relation to safety, a daily intake of 12 g of formulated LEAA supplementation did not induce allergic reactions or affect the liver, kidney, or blood sugar levels. In our trial, we observed the superiority of the LEAA group as increments in muscle density in the radiologic test without protein increments and improvements in general quality of life as measured using questionnaires. In addition, we proved the safety of LEAA supplementation and did not observe any generalized or local adverse or allergic reactions. Further, blood tests showed no hepatorenal toxicity, and glucose levels did not increase.

To date, LEAAs have been used to promote muscle recovery after damage and myofibrillar protein synthesis.^[23]^ A mechanism of muscle damage recovery has been suggested: LEAA can reduce muscle breakdown by decreasing the muscle turnover,^[24]^ and a branched essential amino complex with leucine can have anti-catabolic effects on the muscle fibers.^[25]^ However, the underlying mechanism is still not fully understood. The results of muscle strengthening and control of muscle pain are still controversial.^[26,27]^ These results may vary depending on how the protein is manufactured and according to the mode and time of administration. The relationship between muscular synthesis and LEAAs supplementation is also not fully understood. However, combinations containing whey protein can help muscle synthesis within the immediate 4-hour stage after exercise,^[25]^ and small doses of leucine have been suggested as essential for muscle synthesis in young adults.^[28]^ However, due to gastrointestinal problems and the possibility of allergic reactions, vegetable proteins such as soy protein are more commonly supplemented than LEAAs in older subjects. Importantly, for promoting muscle synthesis and recovery mechanisms, LEAAs should not be ignored, and a combination of soy and whey protein may be highly beneficial. Furthermore, the intake of LEAAs may be more important in elderly patients than in healthy subjects. Whey protein and soy protein have different properties. In general, soy protein has the advantage of a lower probability of allergic reactions, despite the disadvantage of insufficient content of leucine, which is required for protein synthesis.^[29]^ For this reason, a mixture of animal and vegetable proteins has been attempted, but the optimum ratio has not been established yet.^[30]^ In our experience, a mixture ratio of 6:4 is suitable; therefore, we evaluated the suitability of this ratio in this study. Throughout the study duration, we did not observe any allergic reactions, hepatorenal toxicity, or blood sugar elevation. Based on these findings, we can presume that the addition of 12 g and 60% of LEAAs is a safe supplementation strategy.

In the knee arthritis patient, regular indigestion of proteins is difficult due to the poor gastrointestinal function caused by medications, and bone and joint function are decreased and accompanied by poor general condition. Although no increase in serum protein level was observed, muscle density increased, as did the quality of life scores in questionnaires. These results showed the efficacy and superiority of the LEAA supplementation group over the control group. It can be interpreted that LEAAs have the effect of decreasing muscle turnover and increasing processes related to muscle synthesis. However, our findings regarding muscle density were confirmed radiologically and revealed no increments in calf size compared with the control group. It should be noted that this result only reflects the superiority of the LEAAs over the control group, which can be attributed to the effect of the duration of the treatment. A significant improvement was observed after supplementation, but the difference was insufficient and within the range. A trial duration longer than 8 weeks could yield more meaningful results, including increments in muscle size. Laboratory^[31]^ and animal studies^[32]^ have shown better efficacy of muscle synthesis than our study; these findings are expected to be meaningful in future studies.

Knee osteoarthritis is a major issue that worsens the individual life in the aging society. Nevertheless, the goal of treatment is changing. In previous years, arthroplasty for the terminal stage and injection for symptom relief were the main options for medical management. However, due to the development of the industry and medical technology, most patients now receive care and treatment before the terminal stage. Currently, supplementation for strengthening, maintaining, and repairing muscle is gaining more importance than ever before. Supplementation for each part of the knee joint is different. Among the structures of the knee joint, the bone is treated with anti-absorptive medication (bisphosphonate or denosumab) for osteoporosis^[33]^ and vitamin D,^[34]^ which have proven effective. For improving cartilage, the supplementation is focused on glucosamine^[35]^ and chondroitin sulfate.^[36]^ However, the relationship between knee osteoarthritis and muscle has only been studied for promoting muscle strength through exercise. It should be noted that muscle strengthening through exercise alone may not be sufficient, and muscle synthesis under such a regime can be different from the muscle synthesis promoted by a combination of exercise and nutrient supplementation. To date, collagen,^[37]^ vitamin D,^[34]^ and natural extracts rich in antioxidants^[38]^ have shown effectiveness in the muscle of the knee joint, both for symptom relief and for improving quality of life. However, laboratory and radiologic tests were not performed in previous studies, and the risk of liver and kidney damage was not reported. A randomized controlled trial investigating soy protein supplementation showed efficacy for pain alleviation and improvement of life quality,^[39]^ but it did not include radiology or laboratory tests.

There are some ingredients, such as long-chain n-3 fatty-acids found in oily fish or fish oil supplements,^[40]^ and antioxidants,^[37]^ which have shown efficacy in decreasing pain associated with osteoarthritis. However, the mechanisms behind this effect remain unclear, and the long-term efficacy is yet to be proven. Pharmacologic therapy except painkillers, such as statins,^[41]^ and various programmed physiotherapy^[42]^ approaches present possible options for supplementing the management of knee osteoarthritis. The management of knee osteoarthritis cannot be achieved by a single modality; therefore, a holistic approach, combining different interventions, is likely to be more effective in improving the quality of life and slowing the progression of osteoarthritis.

Based on our results, supplementation with LEAA and a combination of whey and soy proteins can promote muscle strengthening in osteoarthritis patients. We suspect that this occurs through several indirect mechanisms, rather than through direct increments in serum protein. Moreover, the lack of effects of increasing serum protein, which will be beneficial to liver, kidneys or in blood sugar levels, is thought to indicate the absence of direct risks. Considering that the protein level in the blood decreases, it can be inferred that 12 g of LEAA increases the utilization rate of the source of protein, which can increase the efficacy and the amount of protein used for building muscle. In addition, supporting enzymes^[43,44]^ and resistance exercise can be beneficial, but an adequate dose of protein has not been determined. Further, our study confirmed an overall improvement in quality of life and physical function, and we expect that continuous supplementation in long-term follow-up studies will show improvement in local function and pain.

Although our research has strengths, there are also some limitations owing to the nature of the study. First, the procedures were not blinded to group allocation, and we did not include a placebo group due to ethical concerns. This is important and needs more research for academic evidence. Further, supplementation with a new combination of proteins may pose risks for the patient, and we opted to use an open-label study design. In this study, we confirmed the safety of this protein supplementation in older subjects. A future study is warranted using a double-blind, randomized controlled design with fewer risks for the participants. Another limitation was the duration of the study, as the long clinical trial period could have affected the results and introduced biases due to other medical conditions. However, the results observed over the 8-week trial showed the superiority of the experimental group over the control group in some parameters. The description of the long-term effects of such administration and the determination of its safety and efficiency require further studies.

We conclude that supplementation with LEAAs in osteoarthritis patients is safe for the liver, kidney, and blood sugar levels, and does not cause allergic reactions. Eight weeks of supplementation with LEAAs is efficacious in increasing muscle density and improving quality of life. The effect of this on pain and joint function is still uncertain and should be determined in a long-term follow-up study. Supplementation with leucine-enriched proteins is safe and efficacious option in the improvement of muscle density and quality of life.

## Author contributions

**Conceptualization:** Seung-Jun Park.

**Investigation:** Chang Hyun Nam, Taehyun Kim.

**Methodology:** Hye Sun Ahn.
